# Helminths of the black-headed gull (*Chroicocephalus ridibundus*) from breeding colonies in north-central Poland

**DOI:** 10.1038/s41598-024-66270-z

**Published:** 2024-07-04

**Authors:** Agata N. Stapf, Izabella Rząd, Katarzyna Królaczyk, Piotr Indykiewicz, Wojciech Gruszka

**Affiliations:** 1 Department of Biological Sciences, Faculty of Physical Culture in Gorzów Wielkopolski, Poznan University of Physical Education, Estkowskiego 13, 66-400 Gorzów Wielkopolski, Poland; 2 Institute of Marine and Environmental Sciences, Faculty of Physical, Mathematical and Natural Sciences, University of Szczecin, Wąska 13, 71-415 Szczecin, Poland; 3 Department of Animal Anatomy and Zoology, Faculty of Biotechnology and Animal Husbandry, West Pomeranian University of Technology in Szczecin, Klemensa Janickiego 33, 71-270 Szczecin, Poland; 4 Department of Biology and Animal Environment, Bydgoszcz University of Science and Technology, Mazowiecka 28, PL-85-084 Bydgoszcz, Poland

**Keywords:** *Chroicocephalus ridibundus*, Cestoda, Digenea, Nematoda, Ecology, Zoology

## Abstract

Among parasites of the digestive tract of the black-headed gull (*Chroicocephalus ridibundus*) in Poland, the best known are species of digenetic trematodes and cestodes. Nematodes of this bird species are not well known. Black-headed gulls, due to their varied diet, migration, life in a flock, and changes of habitat, can become infected with various species of helminths, and like synanthropic birds, they can spread the dispersal stages of parasites across urban and recreational areas. In the present study, an attempt was made to identify the helminth fauna of *C. ridibundus* from breeding colonies in north-central Poland. The aim of the study was to describe the taxonomic structure of parasites of the digestive tract of the black-headed gull and determine the quantitative parameters of their occurrence. A total of 43 black-headed gulls were examined post-mortem for gastrointestinal helminths, resulting in the identification of four cestodes (*Paricterotaenia porosa*, *Lateriporus clerci*, *Anomotaenia micracantha*, and *Wardium fusum*), three trematodes (*Diplostomum pseudospathaceum*, *Plagiorchis laricola*, and *Apophallus muehlingi*), and three nematodes (*Eucoleus contortus*, *Cosmocephalus obvelatus*, and *Porrocaecum ensicaudatum*).* Lateriporus* *clerci* (in adult form), *C. obvelatus* and *P. ensicaudatum* (in larval form) were recorded for the first time in the black-headed gull in Poland.

## Introduction

The parasite fauna of the digestive tract of black-headed gull *Chroicocephalus ridibundus* (L.) is characterized by relatively high species richness. Among the helminth fauna of this bird in Poland, the presence of 14 species of cestodes and as many as 19 species of digenetic trematodes has thus far been confirmed^1^. The nematode fauna of black-headed gulls in Poland is not well known. Only two species of nematodes, *Cyathostoma lari* Blanchard, 1849 and *Eucoleus contortus* (Creplin, 1839), have been recorded in black-headed gulls in Poland^2,3^. Parasitological examination of the droppings of black-headed gulls has been carried out by Indykiewicz and Janiak^4^ who detected nematodes of the genus *Capillaria* spp.

The cestode fauna of the black-headed gull includes parasitic worms belonging to the family Diphyllobothridae, including *Diphyllobothrium dendriticum* (Nitzsch, 1824), *Ligula intestinalis* (Linnaeus, 1758), *L. colymbi* Zeder, 1803, *Schistocephalus solidus* (Müller, 1776), and *S. pungitii* Dubinina, 1959; the family Dilepididae, including *Anomotaenia micracantha* (Krabbe, 1869), *A. hydrochelidonis* Dubinina, 1953, *Lateriporus clerci* (Johnston, 1912), and *Paricterotaenia porosa* (Rudolphi, 1810); and the family Hymenolepididae: *Aploparaksis larina* (Fuhrmann, 1925), *Echinocotyle multiglandularis* (Baczyńska, 1914), *Sobolevicanthus octacanthoides* (Fuhrmann, 1906), *Wardium fusum* (Krabbe, 1869), and *W. cirrosum* (Krabbe, 1869).

Trematodes occurring in the black-headed gull are well known in Poland and around the world, and knowledge concerning them—both faunistic and ecological aspects—is updated on an ongoing basis^1,5–9^. Most trematode species thus far recorded in the black-headed gull in Poland are associated with freshwater ecosystems, belonging to the following families: Diplostomidae (*Diplostomum baeri* Dubois, 1937, *Diplostomum paracaudum* (Iles, 1959) Shigin, 1977 and *Diplostomum spathaceum* (Rudolphi, 1819) Olsson, 1876); Strigeidae (*Ichthyocotylurus erraticus* (Rudolphi, 1809) Odening, 1969, *I. pileatus* (Rudolphi, 1802) Odening, 1969, *I. platycephalus* (Creplin, 1825) Odening, 1969, and *I. variegatus* (Creplin, 1825) Odening, 1969); Echinostomatidae (*Echinoparyphium clerci* Skrjabin, 1915, *E. nordiana* Baschkirova, 1941, *E. recurvatum* (von Linstow, 1873) Lühe, 1909), and *Echinostoma revolutum* (Fröhlich, 1802) Looss, 1899); Heterophyidae (*Apophallus muehlingi* (Jägerskiöld, 1899) Lühe, 1909, Schistosomatidae (*Gigantobilharzia mazuriana* Khalifa, 1974 and *G. monocotylea* Szidat, 1930); Plagiorchiidae (*Plagiorchis laricola* Skrjabin, 1924 and *P. moravicus* Sitko 1993); Prosthogonimidae (*Prosthogonimus ovatus* (Rudolphi, 1803) Lühe, 1899; and Eucotylidae (*Tanaisia fedtschenkoi* Skrjabin, 1924)^1^.

The ecology of the black-headed gull, including its diet, foraging and breeding habitats, and migration, is conducive to the acquisition of internal parasites. The food of these omnivorous birds consists mainly of invertebrates, and thus potential intermediate hosts for helminths, primarily earthworms and insects (e.g. Scarabaeidae, Carabidae, Chrysomelidae, Staphylinidae, Tipulidae, Culicidae, Simuliidae, Ephemeroptera, Trichoptera and Odonata), molluscs (terrestrial species—keelback slugs of the family Limacidae and roundback slugs Arionidae—and freshwater species *Cardium* sp., *Tellina* sp., and *Bithynia* sp.), seeds, fruits, and carrion, as well as food produced by humans. The black-headed gull breeds mainly on islands at various types of water bodies: dam reservoirs, natural lakes, fish ponds, gravel pits, or rivers. The species nests at high densities, and colonies often comprise more than 100 pairs^10,11^. Black-headed gulls are long-lived birds, with an adult annual survival rate of 0.90^12^ and a maximum recorded age of 33 years^13^. Their reproductive output is usually about 1.0–1.5 fledglings per breeding pair^14^, making adult survival one of the major components of population dynamics^15^. Black-headed gulls from breeding colonies in Poland winter in Western and Southern Europe^16,17^. Black-headed gulls are believed to contribute to the spread of parasites which can pose a threat to the health of companion animals (cats and dogs), farm animals, and humans^4^.

In this paper, an attempt was made to identify the helminth fauna of *C. ridibundus* from breeding colonies situated in north-central Poland. The aim of the study was to describe the taxonomic structure of parasites of the digestive tract of the black-headed gull and determine the quantitative parameters of their occurrence.

## Materials and methods

The material for the research consisted of nematodes, trematodes and cestodes isolated from the gastrointestinal tracts of 36 adult black-headed gulls. Parasitological examination was performed in 2017–2019 on 43 black-headed gulls found dead in breeding colonies situated in north-central Poland, in the Kuyavian–Pomeranian Voivodeship, on small islands in lakes Kusowskie, Koronowskie, and Pakoskie, as well as on the River Brda flowing through Bydgoszcz and on an island in the active gravel pit Skoki Duże, a Natura 2000 protected area for birds. A detailed description of the islands and the size of the colonies are presented in works by Kitowski et al.^18^, Jakubas et al.^19^ and Indykiewicz et al.^20^. Parasites were identified using standard methods, including fixation, staining, and preparation of microscope slides. Cestodes were fixed in 75% ethyl alcohol, and trematodes and nematodes in 70% ethyl alcohol. Cestodes were stained with iron-acetocarmine prepared according to the formula given by Georgiev^21^. Slides of selected cestode specimens were prepared in Hoyer’s medium^22^. Trematodes were stained with carmine alum, cleared in clove oil, and mounted in Canada balsam^23,24^. Unfixed nematode specimens cleared in glycerine were examined under a microscope^24^.

Helminth species were identified using available keys and numerous original works. The taxonomic affiliation of cestodes was presented according to Pojmańska and Cielecka^25^ and Pojmańska et al.^1^. Species identification of trematodes was carried out using keys given by Niewiadomska^26^, Niewiadomska and Pojmańska^27^, Yamaguti^28,29^, Gibson et al.^30^, Jones et al.^31^, and Bray et al.^32^, as well as original works by Bychovskaja-Pavlovskaja^33^, Niewiadomska^34^, and Sonin^35^. The taxonomic affiliation of nematodes was determined according to Baruš and Sergeeva^36^, Stapf et al.^37^, Kim et al.^3^, McNeill and Anderson^39,40^ and Smogorzhevskaya^41^. The taxonomic affiliation of 17 cestode and 24 trematode specimens was not determined, and 39 nematode specimens were identified only to subfamily. This was due to severe damage to the bodies of the parasites, most likely resulting from the substantial degree of decomposition of several of the birds. Quantitative parameters of the occurrence of parasites, i.e. prevalence and intensity of infection (mean intensity and range of intensity), were calculated according to definitions given by Bush et al.^42^ In this study, prevalence refers to the percentage of infected gulls among all tested gulls; mean intensity refers to the number of parasites divided by the number of infected gulls; and range of intensity refers to the minimum and maximum number of parasites infecting a single gull.

A statement confirming the permission for collecting the dead birds: Resolution of the Local Bioethical Commission for Experiments on Animals in Bydgoszcz No 13/2017 of 21.03.2017 and Łódź No 40/ŁB66/2017 of 05.06.2017 and Decision of the Regional Environmental Protection Directorate in Bydgoszcz No WPN.6401.1.118.2017.RS of 03.04.2017, WPN.6401.1.96.2017.MP of 24.07.2017, and WPN.6401.11.105.2018.RS of 06.04.2018.

All experiments were performed in accordance with relevant guidelines and regulations.

## Results

The prevalence of infection in the gulls was 83.7% (36 gulls infected with helminths among 43 examined). A total of 530 specimens of all helminth species were collected.

There were 45 cestode specimens isolated from the digestive tracts of gulls. Four cestode species were recorded: *Paricterotaenia porosa* (Rudolphi, 1810), *Lateriporus clerci* (Johnston, 1912), *Anomotaenia micracantha* (Krabbe, 1869) (Family Dilepididae), and *Wardium fusum* (Krabbe, 1869) (family Hymenolepididae) (Table 1).

**Table 1:** Prevalence and intensity of helminth infection in black-headed gulls.

	Number of infected gulls	Prevalence [%]	Mean intensity	Range of intensity
Cestoda				
*Paricterotaenia porosa*	3	6.97	1.66	1–2
*Lateriporus clerci*	4	9.30	2.75	1–6
*Anomotaenia micracantha*	1	2.32	7	7
*Wardium fusum*	1	2.32	5	5
Cestoda spp.	6	13.95	2.83	1–4
Total	12	27.9	3.75	1–7
Digenea				
*Diplostomum pseudospathaceum*	6	13.95	49.0	7–170
*Plagiorchis laricola*	6	13.95	3.0	1–7
*Apophallus muehlingi*	1	2.32	1.0	1
Digenea spp.	3	6.97	8.0	3–12
Total	11	25.58	38.4	5–174
Nematoda				
*Eucoleus contortus*	15	34.8	7.26	1–17
*Cosmocephalus obvelatus*	1	2.32	1	1
*Porrocaecum ensicaudatum*	9	20.9	2.1	1–5
Capillariinae spp.	9	20.9	4.33	2–7
Total:	26	60.46	6.46	1–17

Eleven gulls were infected with trematodes, which were isolated from the digestive tracts. Three trematode species were recorded: *Diplostomum pseudospathaceum* Niewiadomska 1984 (Family Diplostomidae), *Apophallus muehlingi* (Jägerskiöld, 1899) (family Heterophyidae), and *Plagiorchis laricola* Skryabin, 1924 (family Plagiorchiidae) (Table 1). *D. pseudospathaceum* was clearly dominant in terms of abundance (number of individuals, intensity of infection)*.*

**Figure 1:**
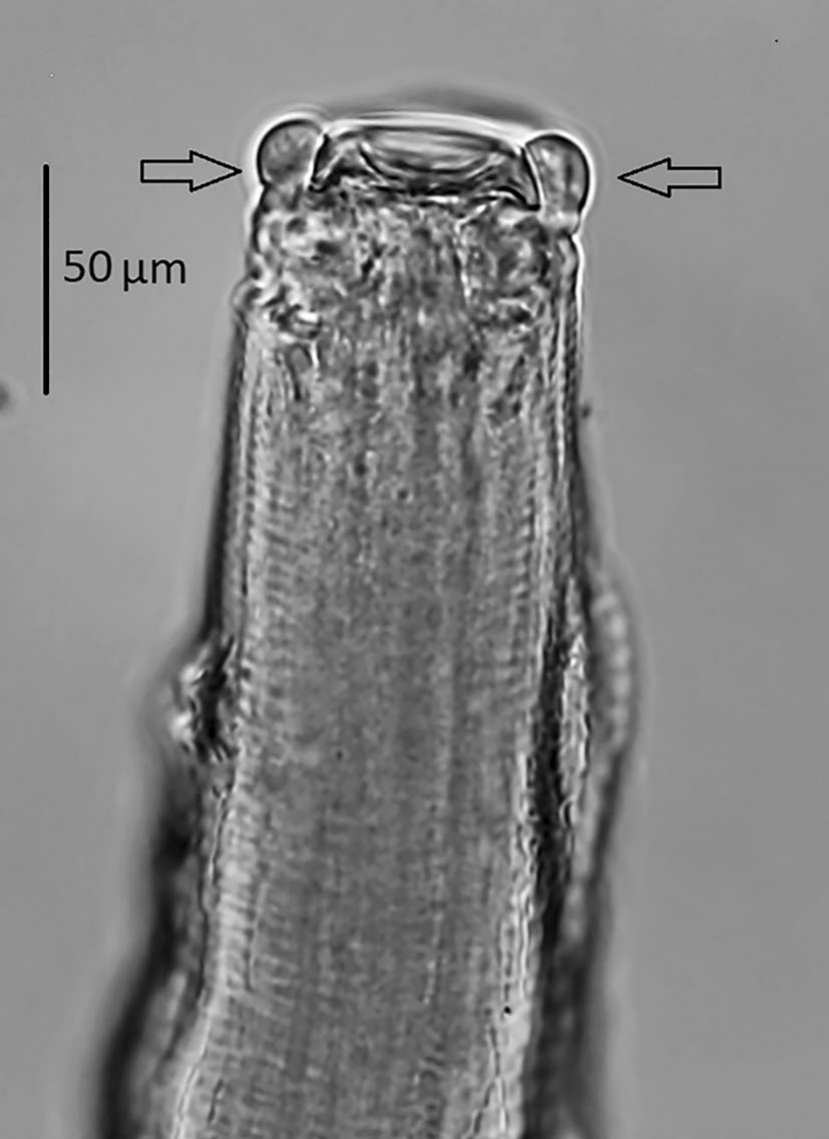
The anterior end of the body of the *Porrocaecum ensicaudatum* larva. The arrows indicate the ring of cuticle, visible on the optical microscope slide as two globules situated on each side of the anterior end.

**Figure 2:**
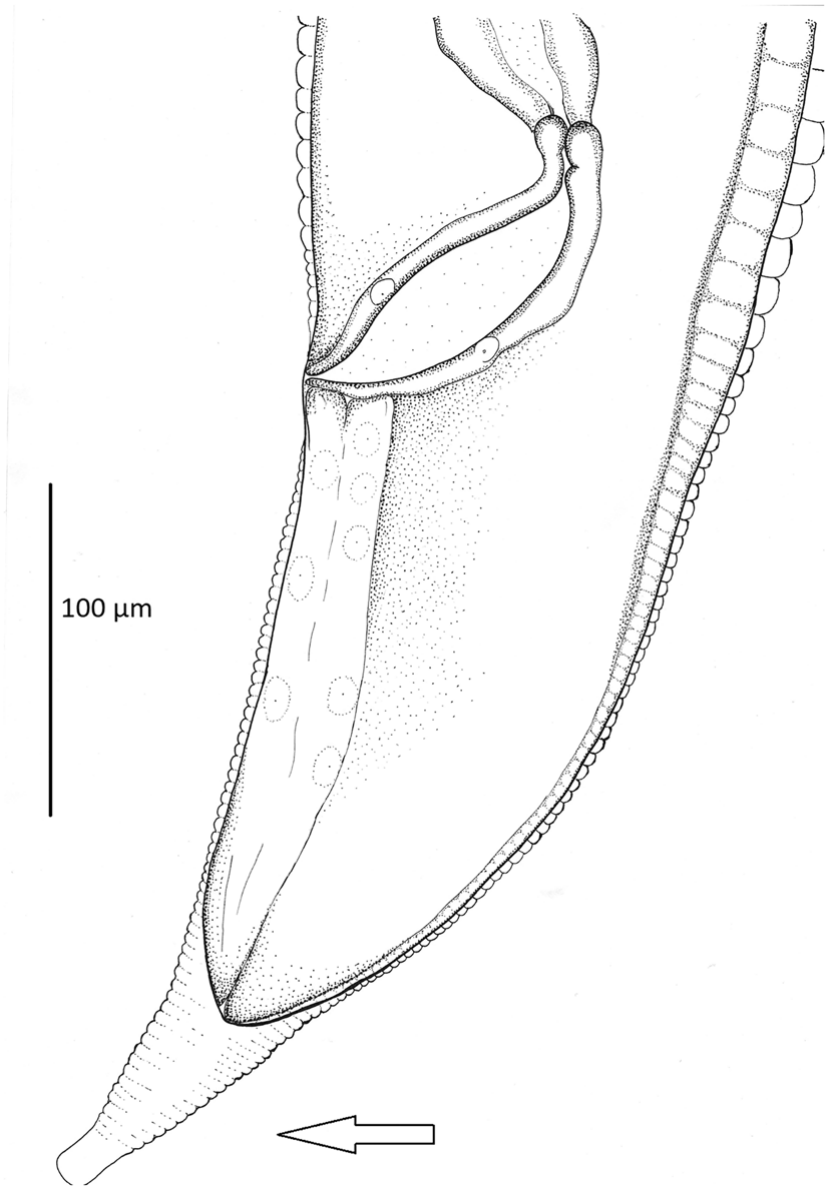
The posterior end of the body of the *Porrocaecum ensicaudatum* larva. The arrow indicates the conical tail.

There were 26 black-headed gulls infected with nematodes. The isolated nematodes were assigned to three species: *Eucoleus contortus* (Creplin, 1839), *Cosmocephalus obvelatus* (Creplin, 1825) Seurat, 1919, and *Porrocaecum ensicaudatum* (Zeder, 1800) (Table 1). Nematodes of the species *E. contortus* and those identified to the subfamily Capillariinae spp. colonized the oesophagus of the hosts. *C. obvelatus* was isolated from the proventriculus. All *P. ensicaudatum* specimens were in the third larval stage and located under the stratum corneum at the site where the proventriculus passes into the gizzard. The first, highly characteristic morphological character of the larvae, making them easy to spot on the background of the dissected gizzard, is a black intestine visible through the colourless body. The anterior segment of the body is rounded. The three labia around the mouth typical of sexually mature forms are absent. However, there is a smooth ring of cuticle, visible on the optical microscope slide as two globules situated on each side of the anterior end (Fig. 1). In some specimens, a cap of arcadial tissue was observed below the mouth. Inside the body of the larvae, an oesophagus and a ventriculus could be seen. Their dimensions and those of other parts of the body are presented in Table 2. The posterior end of the body terminates in a conical tail (Fig. 2). There are no male or female sex organs.

**Table 2:** Morphological characters of *Porrocaecum ensicaudatum* larvae [mm].

Measurement	Oswald^96^	Okulewicz^97^	Own material
Body length	3.60–4.63	2.987–3.867	3.0–3.57
Body width	0.097–0.148	0.082–0.103	0.075–0.098
Oesophagus length	0.379–0.462	0.365–0.437	0.28–0.49
Ventriculus length	0.106–0.129	0.078–0.112	0.06–0.125
Ventriculus width	0.055–0.070	–	0.034–0.055
Tail length	0.117–0.153	0.108–0.154	0.114–0.154

## Discussion

Among the helminth fauna of the black-headed gull, nematodes were the group with the highest prevalence, followed by cestodes, while the fewest birds were infected with trematodes. This pattern of prevalence is in agreement with the findings of Indykiewicz and Janiak^4^.

This is the first record of an adult specimen of the species *Lateriporus clerci* (Johnston, 1912) in Poland. Gulls are definitive hosts of this parasite, while amphipod crustaceans of the genus *Gammarus* function as intermediate hosts^25^. The life cycle of the cestode may also include ducks as a paratenic host. It has been recorded in Europe and Asia, e.g. in *C. ridibundus*, *Larus canus*, and *Larus argentatus* ssp. *mongolicus* from Lake Baikał in south-eastern Siberia, as well as in *Chroicocephalus philadelphia* from Cooking Lake in Alberta, Canada^43,44^. In Poland, Czapliński et al.^45^ detected larvae of this cestode in *Gammarus pulex* in the Masurian Lake District.

The black-headed gull is a definitive host for all cestode species recorded in the study. The species identified in the research material had previously been recorded (except for *L. clerci*) among the cestode fauna of these birds in Poland and other parts of Europe^1,46,47^. The material was not confirmed to include cestodes of the genus *Hymenolepis*, whose eggs were previously found in 60 adult black-headed gulls from breeding colonies on an island in a lake in Myślęcinek, in the Kuyavian-Pomeranian Voivodeship^4^.

*Paricterotaenia porosa* (syn *Choanotaenia porosa* Rudolphi, 1810) is a widespread parasite in Europe, Asia, and North and Central America. Mature specimens have been recorded in the black-headed gull, common gull, great black-backed gull, and common tern. The intermediate host in its life cycle is unknown^25^. In Poland, cestodes of this species have been identified in gulls on the Baltic Coast^48,49^, in the Masurian Lake District^50,51^, in the Wielkopolsko-Kujawska Lowland^52,53^, and in eastern Poland^54^. In Europe, *P. porosa* has been recorded in *Chroicocephalus ridibundus* in Denmark (in 16 gulls among 111 examined)^55^ and in northern Karelia in Russia (7 cestodes in one black-headed gull)^5^. In Great Britain, cestodes identified only to genus as *Paricterotaenia* sp. were detected only in a single five-day-old black-headed gull chick^56^.

Gulls and terns function as definitive hosts of *Anomotaenia micracantha* (syn *Anomotaenia micracantha micracantha* Krabbe, 1869). Its intermediate host is unknown. It has been recorded in Europe and North America^25^. Cestodes of this species have been recorded in Poland in the black-headed gull and black tern in the Masurian Lake District^50^ and the Wielkopolsko-Kujawska Lowland^53^.

Gulls are a definitive host of *Wardium fusum*, syn. *Aploparaksis fusus* (Krabbe, 1869), and the intermediate host is the crustacean *Artemia salina*^25^. The species has been recorded in Europe and Asia, and in Poland in the black-headed gull on the Baltic Coast^48,49^ and in the Wielkopolsko-Kujawska Lowland^53^.

The black-headed gull is a host for all species of trematodes recorded in the study. Trematodes are common parasites of black-headed gull, recorded earlier in this bird in Poland, with broad host specificity^1^. *Diplostomum* *pseudospathaceum*, *Plagiorchis* *laricola* and *Apophallus* *muehlingi* are associated through their life cycles with freshwater environments. The fact that these trematodes were recorded in a synanthropic environment in the region of the Vistula is indicative of foraging by gulls in the inland ecosystem and of a diet consisting in part of natural food which included the hosts of trematodes, which were present in them in larval form (metacercariae). These include fish, insect larvae, crustaceans, and molluscs. This finding indirectly demonstrates the occurrence of invertebrates in the foraging grounds of gulls—snails of the genera *Lymnaea*, *Stagnicola*, and *Lithoglyphus*, which in the life cycles of trematodes are hosts of parthenogenetic forms of trematodes. The three trematode species identified in the material have also been recorded in black-headed gulls in the Czech Republic^7^. In northern Karelia in Russia, as well as in Great Britain and Denmark, the occurrence of other trematode species belonging to the same genera as those recorded in the present study has been described^5,55,56^.

Birds of the family Laridae can be parasitized by several species of the genus *Diplostomum*^57–60^. Pojmańska et al.^1^ stress that some published data on *Diplostomum spathaceum* may in fact refer to *D. pseudospathaceum*, as in the past these two species were not distinguished^34^. Cercariae of *D. pseudospathaceum* develop in the great pond snail *Lymnaea stagnalis* and the marsh pond snail *Stagnicola palustris*. Metacercariae are found in the eye lens of many species of fish, including cyprinids and percids^26^. *Diplostomum* *spathaceum* has often been recorded in fish in Poland, but according to Niewiadomska^61^, it is a species complex which includes *D. pseudospathaceum*. It is difficult to distinguish *Diplostomum* species on the basis of the morphology and morphology of metacercariae due to their close similarity and individual variation. Adult forms of these trematodes do not exhibit such high variation of taxonomic characters as the metacercariae. In a study by Morozińska-Gogol^62^, in order to determine the *Diplostomum* species found in three-spined stickleback *Gasterosteus aculeatus* on the Baltic coast, black-headed gulls were experimentally infected with these metacercariae. The adult trematodes obtained in this manner were predominantly identified as *D. spathaceum*, indicating that this trematode is the most numerous parasite of the eye of three-spined stickleback in the Baltic^62^.

*Plagiorchis laricola* is a widespread species and is found in many species of birds, including storks, plovers and gulls. The host for the sporocysts and cercariae are the great pond snail and the wandering snail *Radix balthica*, and the metacercariae develop in insect larvae, crustaceans, and molluscs^27^.

*Apophallus muehlingi* is a parasite of piscivorous birds. In Poland it has been recorded in gulls of the genus *Larus* and *Gavia stellata*. It has also been found in some mammals that feed on fish^1^. The host of the rediae and mother sporocysts is the gravel snail *Lithoglyphus naticoides*. The metacercariae develop in cyprinid fish^63^. The role of the gravel snail as a host is confirmed by the results of research and analyses by numerous authors^64–67^. In Poland, larval forms of *A. muehlingi* occurring in gastropods have not been described. The gravel snail is an alien species in Poland, from the Pontic-Caspian steppe region. At the same time it is one of the endangered species listed in the Polish Red Book of Animals. Its occurrence has been described at many river sites in Poland, including the Vistula^68,69^. In the light of current knowledge of the role of *L. naticoides* in the life cycle of the trematode *A. muehlingi*, this snail can be said to contribute to the persistence of the *A. muehlingi* population in the area where the gulls for the research were obtained.

The presence of two nematode species has thus far been recorded in Poland: *Cyathostoma lari* and *Eucoleus contortus*^1^. The present study confirmed the occurrence of *E. contortus* (subfamily Capillariinae), and additionally nematodes *Cosmocephalus obvelatus* and *Porrocaecum ensicaudatum* were detected*.* The presence of *C. obvelatus* is the first recorded case in the gull *C. ridibundus* in Poland. This species is found in gulls and other birds associated with the aquatic environment (e.g. *Mergus serrator*, *Sterna arctica*, *Gavia arctica*, *Podiceps cristatus*, and *Alca torda*)^38,41,70^. Adult forms parasitize the oesophagus or proventriculus of the definitive host. The life cycle includes an intermediate host—invertebrates of the order Amphipoda (genera *Gammarus*, *Hyalella*, and *Crangonyx*)^71^. In Poland, the presence of this nematode species has been described in *Phalacrocorax carbo sinensis*^1^.

*Porrocaecum ensicaudatum* in the form of third-stage larvae is the first confirmed case in the black-headed gull in Poland. Sexually mature nematodes parasitize birds of the order Passeriformes. They develop to the invasive L3 larval form in the intermediate host, which is earthworms^39^. In birds of the order Charadriiformes (the genus *Larus*), the development of L3 larvae is inhibited. Birds of the species *C. ridibundus* are considered to be atypical hosts. *P. ensicaudatum* larvae live under the stratum corneum at the site where the proventriculus passes into the gizzard. A morphologically similar species for which the black-headed gull and other birds of the order Charadriiformes are recognized as definitive hosts is *P. semiteres*. Both of these nematode species can parasitize the definitive host in the invasive larval stage. The two species can be distinguished by calculating the ratio of the ventriculus length to the intestinal caecum length^70,72,73^. The length of the caecum of *P. ensicaudatum* larvae is no more than half the length of the ventriculus^74^. Analysis of measurements of the length of the intestinal caecum and ventriculus reveals a ratio of 1:0.29–0.35. The results are similar to the values obtained by Supryaga and Supryaga^75^.

*E. contortus* had the highest prevalence and intensity among all recorded nematodes. Indykiewicz and Janiak^4^ also reported a predominance of nematode eggs of the genus *Capillaria* (subfamily Capillariine) in the droppings of black-headed gulls in comparison to other groups of helminths. Infection of gulls with nematodes *E. contortus* confirms the presence of earthworms in the diet of these birds. Earthworms (Oligocheta) are intermediate hosts of nematodes of the genera *Capillaria* and *Eucoleus*^37,70^. The black-headed gull is a typical host for the nematode species found in the present study. Their presence has been described in the black-headed gull in countries such as the Czech Republic^73^ and Great Britain^76^.

As colonial birds, gulls have higher species richness of parasitic worms than non-colonial birds^77^. The species richness of the helminth fauna of birds of the order Charadriiformes is primarily determined by the habitat (freshwater and/or marine) and diet (omnivory, invertebrates, fish-based diet)^78^. Black-headed gulls are hosts for nearly 200 species of parasitic worms, with trematodes believed to have the highest species richness (120 species detected) (London Natural History Museum host-parasite database^79^). This is confirmed by research by Gutiérrez et al.^78^. The authors noted that the species richness of trematodes is higher in birds whose diet includes mixed food sources (invertebrates and fish) or in omnivorous birds occupying marine or freshwater/marine habitats. The number of species of nematodes, cestodes and acanthocephalans is not influenced by the host diet or habitat, but in the case of nematodes, they predominate over other helminths in the parasite fauna of birds which are habitat generalists. The number of trematode species also increases with geographic range, while the species richness of cestodes increases with geographic latitude. The species richness of nematodes decreases as migration distance increases^78^.

The helminth fauna of black-headed gulls *C. ridibundus* from several areas of Russia, i.e. northern Karelia, the Republic of Mordovia, and Lake Baikal in south-eastern Siberia, has been studied during the last 10 years^5,44,80^. Dorzhiev et al.^44^ examined nearly the same number of gulls as in the present study. While the total prevalence of helminths in black-headed gulls from Lake Baikal was 79.5%, in the present study it was more than 83%. There are considerable differences in the number of species of parasitic worms. Gulls from Lake Baikal were infected with 11 species of cestodes, 23 species of trematodes, 10 species of nematodes, and 3 species of acanthocephalans. In comparison with these data, the species richness of parasites of black-headed gulls from breeding colonies in north-central Poland seems quite modest: 4 species of cestodes, 3 species each of trematodes and nematodes, and no acanthocephalans. However, the species composition of helminths in gulls from these regions overlaps to only a small extent, mainly in the case of cestodes^44^. No nematodes were found in gulls from the Republic of Mordovia in Russia, but the birds were infected with two species of cestodes, which were also found in the material in the present study, and 4 species of trematodes, of which one, *Plagiorchis laricola*, was present in gulls from Poland. The helminth fauna of black-headed gulls from North Karelia was poorer in species than in the material analysed in the present study. A species common to both regions was *Paricterotaenia porosa*.

Other Laridae species in which similar species richness of helminths has been described are the European herring gull *Larus argentatus* and *Larus argentatus* ssp*. Mongolicus*^44,79,81,82^. The abundance of parasite species can be explained by the range of occurrence of these birds and their type of diet. In seagulls from both the Kola Bay of the Barents Sea and Lake Baikal, trematodes were represented in the highest numbers, with fewer cestodes and nematodes^44,81,82^.

Gulls *Chroicocephalus ridibundus*, like *Larus argentatus*, can be hosts of cestodes *Diphyllobothrium dendriticum*, which pose a threat to human health. Infections with *D. dendriticum* in humans still receive little attention, due to their sporadic occurrence. However, they are an example of a food-borne zoonosis and a consequence of the globalization of the fish trade, transport of fresh fish on ice, increased human migration, and changes in culinary habits. Recent cases of infection with *D. dendriticum* in the Netherlands, Switzerland and the Czech Republic indicate the need to address factors spreading parasites which are rare or do not belong to the native fauna^83^. The gulls mentioned above can also be hosts of the cestode *Ligula intestinalis*, which parasitizes cyprinid fish in the form of plerocercoid larvae^44,83–85^. This parasite does not pose a threat to humans.

Helminth species were precisely identified in the present study, but there were certain limitations, e.g. the poor state of some of the carcasses. Nevertheless, in addition to the fresh carcasses examined, birds that were not fresh were used as well, and it was possible to obtain helminths from them in accordance with literature recommendations^86–89^. In future endeavours, the use of molecular methods in addition to morphological methods would be of value. Molecular methods can be useful in studies of cryptic species. For example, the use of genetic methods has proven useful in identifying the cryptic diversity of *Diplostomum* species, including species identification of specimens obtained from intermediate and definitive hosts^90–93^. Researchers studying *Plagiorchis* sp. provide molecular evidence of the high diversity of the genus in snails and stress the need to use molecular methods in further study of this diversity, as well as the importance of further molecular testing to establish the links between various stages in the life cycle of these parasites^94,95^.

## Conclusion

The results of the present study contribute new information regarding helminths of the black-headed gull. Given the new information about the parasite species found in black-headed gull and the dependency of their occurrence on the diet of these birds and their foraging grounds, parasitological research should be continued.

## Data Availability

Te datasets used and analyzed during this study are available from the first author (A.S.) upon reasonable request.
